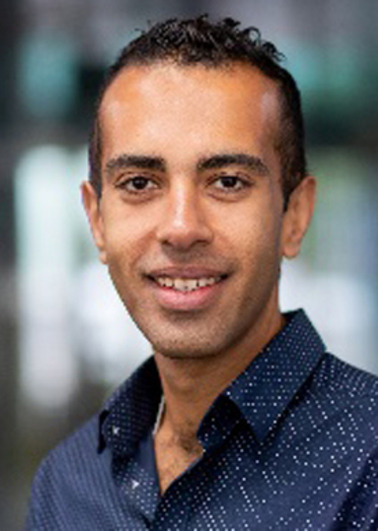# Achieving Control in Micro‐/Nanomotor Mobility

**DOI:** 10.1002/anie.202214754

**Published:** 2022-12-14

**Authors:** Alexander D. Fusi, Yudong Li, A. Llopis‐Lorente, Tania Patiño, Jan C. M. van Hest, Loai K. E. A. Abdelmohsen

**Affiliations:** ^1^ Departments of Chemical Engineering and Chemistry, and Biomedical Engineering Institute for Complex Molecular Systems Technische Universiteit Eindhoven Het Kranenveld 14 5612 AZ Eindhoven The Netherlands; ^2^ CIBER de Bioingeniería, Biomateriales y Nanomedicina (CIBER-BBN) Institute of Molecular Recognition and Technological Development (IDM) Universitat Politècnica de València Camino de Vera s/n 46022 Valencia Spain

**Keywords:** Directionality, Mobility, Multi-Mode, Quorum, Rotation

## Abstract

Unprecedented opportunities exist for the generation of advanced nanotechnologies based on synthetic micro/nanomotors (MNMs), such as active transport of medical agents or the removal of pollutants. In this regard, great efforts have been dedicated toward controlling MNM motion (e.g., speed, directionality). This was generally performed by precise engineering and optimizing of the motors′ chassis, engine, powering mode (i.e., chemical or physical), and mechanism of motion. Recently, new insights have emerged to control motors mobility, mainly by the inclusion of different modes that drive propulsion. With high degree of synchronization, these modes work providing the required level of control. In this Minireview, we discuss the diverse factors that impact motion; these include MNM morphology, modes of mobility, and how control over motion was achieved. Moreover, we highlight the main limitations that need to be overcome so that such motion control can be translated into real applications.

## Graphical abstract

This Minireview highlights the advancements in the area of micro‐ and nanomotors and discusses current methods and mechanisms to control their movement. We review motors’ design, modes of propulsion and incorporation of different modes in one motor chassis so that control can be achieved.

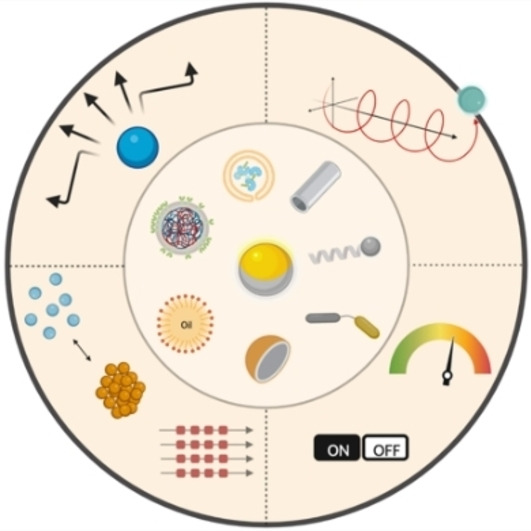

## 1 Introduction

Artificial micro‐/nanomotors (MNMs) are highly valuable instruments to attain both a better fundamental understanding of cell motility and to expand the horizon of active materials applications. Inspired by living organisms, MNMs convert different sources of energy into mechanical work, leading to translational or rotational movement. Their design for artificial motion can elucidate the complexities and influencing factors of naturally found cell motion, which can subsequently be applied for medicinal, environmental, or fundamental purposes.

**Figure 1:**
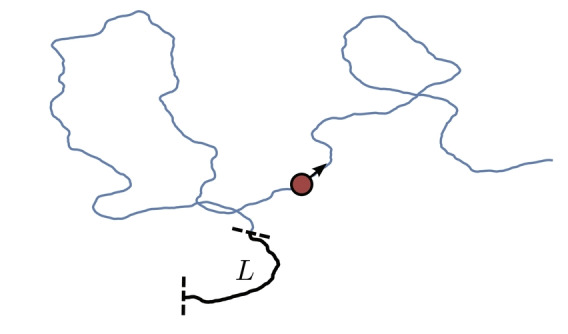
Motion trajectory of active Brownian particles with loss of directionality influenced by rotational diffusion. Reproduced from Ref. [3] under CC BY 3.0 license.

At the micron/nano scales, and low Reynolds number hydrodynamics, inertial forces are nominal, and viscosity greatly impacts MNMs’ motion—excluding conventional, reciprocal (e.g., flapping arms) macroscale motion tactics.^[1]^ Moreover, Brownian motion and rotational diffusion resulting from thermal fluctuations represent challenging aspects for precise motion control of MNMs (Figure 1).[^2^, ^3^] Overcoming such challenges requires asymmetry‐dependent, non‐reciprocal motion, facilitated by motor design and actuation modes. This review aims to discuss motor design, the physical basis of available swimming mechanisms and actuation modes, and how to achieve mobility control. Given the vast array of available propulsion mechanisms and motion controls, we will focus on informative examples of the different concepts rather than attempting to be comprehensive. We would like to refer to more in‐depth reviews about topics discussed in this article for those interested.[^4^, ^5^, ^6^, ^7^, ^8^, ^9^, ^10^] Furthermore, as the main application of MNMs is in the biological realm, we will also discuss the potential usage of different systems from a biomedical perspective.

## 2 Morphology of Micro‐/Nanomotors

**Figure 2:**
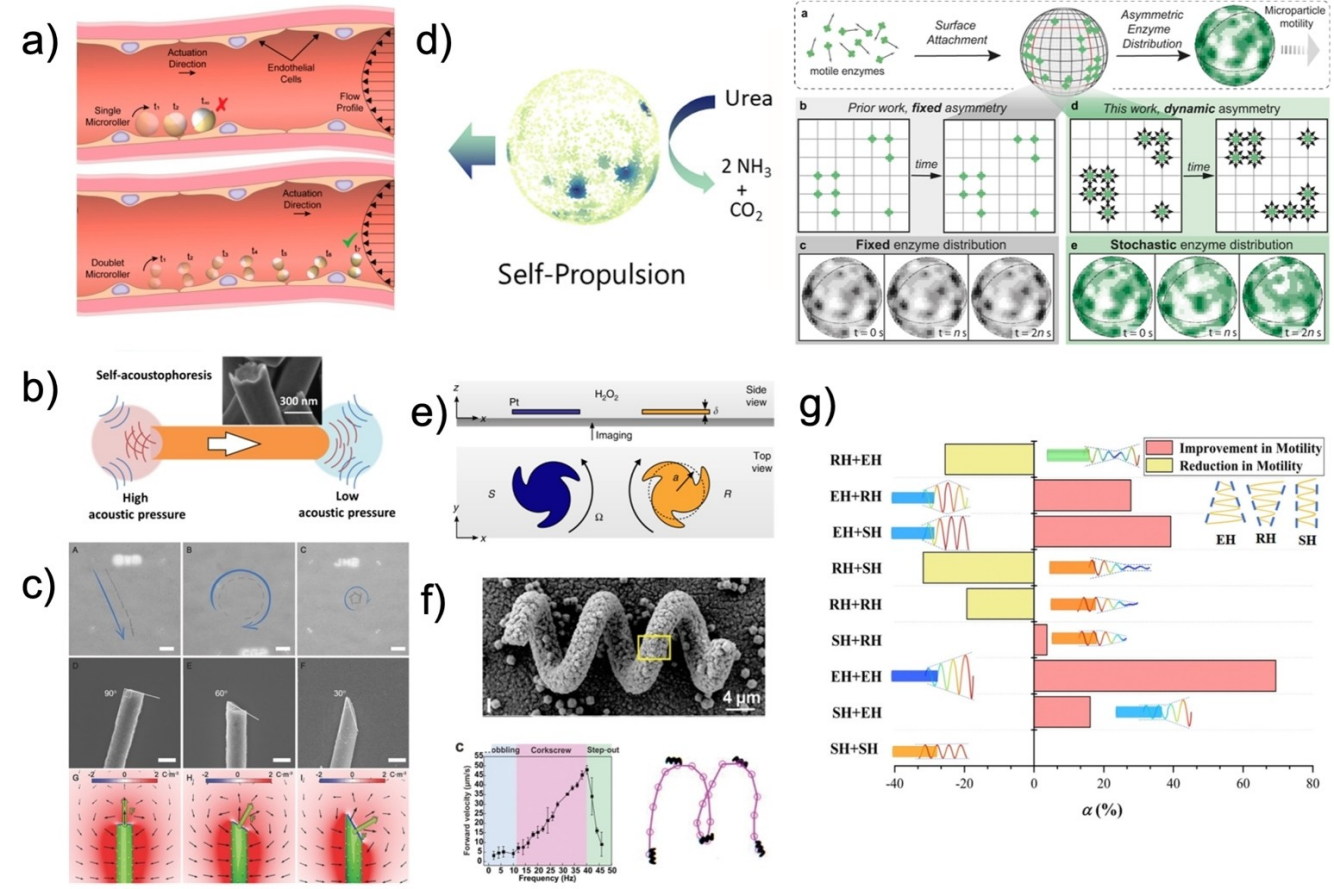
(a) Janus motor in blood vessels and dimerization for optimal mobility on microtopographies. Adapted from Ref. [11]. (b) Proposed mechanism for activating acoustophoresis in asymmetric metallic microrods. Adapted from Ref. [17]. (c) Motion trajectory and numerical simulation of directed charge distribution of silicon nanowires, with a terminal miter angle morphology. Reproduced from Ref. [19]. (d) Fixed and stochastic enzyme surface heterogeneity induced propulsive motion. Adapted from Ref. [22, 23]. Ref. [23] is under the terms of the CC BY 4.0 license. (e) Directed rotation of particles by shape anisotropy, axial symmetry, and fore‐and‐aft violations. Adapted from Ref. [14] under the terms of the CC BY 4.0 license. (f) Metal‐organic framework helical artificial flagellum with magnetic field induced corkscrew motion. Adapted from Ref. [24]. (g) Motion dynamics based on hierarchical tail design of magnetically driven MNMs. Reproduced from Ref. [25].

Mobility is imparted by the shape anisotropy and surface asymmetry of MNMs. Variations in surface composition and shape provide tools to direct motion and circumvent hydrodynamic issues. Furthermore, this also allows MNMs to navigate effectively in complex environments such as blood vessels or intestines (Figure 2a).^[11]^

Regarding anisotropy, the first consideration for mobility comes from the design of geometrical design, aspect ratio, and morphology of motor active sites, which affect motion and transport within fluids.[^12^, ^13^] In principle, the concave or convex curvature of the active site generates distinctive distributions of potential energy that induce a build‐up of local concentration or dissipation of the stored energy of the system. Thereafter, the system equilibrates toward an entropic maximum through compensating fluid flows, which propel MNMs in a continuous phase.^[14]^ With these thermodynamic processes in mind, concave active sites concentrate the energy to a focal point near the curvature that induces directed flows of the fluid or its release as concentrated bursts, as observed with bowl‐shaped (stomatocyte) or metallic rod motors.[^15^, ^16^] Alternately, convex curvatures scatter the energy radially, decreasing the density of the potential energy near the active site (Figure 2b).[^15^, ^17^] It is important to note that considerations for the curvature of the active site may influence propulsion speeds, and motion is more dependent on overarching motor asymmetry and actuation mode, e.g., the case of asymmetric motor oscillation and motion according to acoustic streaming models.^[18]^ Finally, the angle and direction of the active site will control the trajectory of the force dissipation. For example, a nanowire end morphology, or generally the miter angle, will create an uneven energy distribution and ratio between the perpendicular and parallel forces of propulsion acting on the particles, inducing circular or even rotational trajectories (Figure 2c).^[19]^

Final considerations in MNM design come from its shapes and symmetry violations. Asymmetrically functionalized surface is necessary to impart mobility above the level of enhanced Brownian motion and can confer, in some cases, controlled directionality, especially for micron‐sized particles.[^20^, ^21^]

An isotropic structure dictates no bias for the direction of fluid fields generated by the active sites and will only result in slightly enhanced Brownian motion. In contrast, fixed or transient asymmetrically located functional sites direct fluid flows and forces in specific directions. Exemplary options of surface asymmetry have been studied through enzymatically powered motors by Patino and Song et al. In both studies, surface heterogeneity either from a fixed surface asymmetry, or stochastic transient enzymatic clusters induced propulsive motion (Figure 2d).[^22^, ^23^] Nonetheless, design considerations with additional symmetry violations, are necessary for achieving specific motion, such as rotational control.[^14^, ^26^]

As Brooks et al. showed, pinwheel‐shaped platinum micromotors can collectively uni‐rotationally move around their axis fueled by hydrogen peroxide (H_2_O_2_). The rotational direction is given by the electrokinetic flow dictated by the asymmetry of the curved fins; this behavior was ascribed to the emergence of OH^−^ producing cathodic regions and water and molecular oxygen‐anodic producing regions. In the cathodic region, the first half‐reaction of H_2_O_2_ decomposition creates an ionic charge imbalance, which induces electrochemical and fluid flows interfacial to the particles, from the high‐curvature edges to the low‐curvature faces (Figure 2e).

Another example of symmetry violations for directed movement is shown with helical nanopropellers of Wang et al. Helically structured wires are magnetized in a field directed along the normal of the long face of the helix. The magnetic moment of the motor can be strongly coupled with rotating magnetic fields, making the particle rotate around its long axis, resulting in translational diffusion within the magnetic field, tuned by the helical pitch and sense of rotation (Figure 2f). The rotational drag coefficient resulting from the structural geometry leads to more efficient rotation around their long axis and subsequent propulsion of the rotating structure.^[24]^ Increased propulsion efficiency can be achieved by including a “head” and tuning the helix shape from a cylinder to an emanative cone. The heads increase the stability of the movement, while emanative tails increase thrust and torque forces at the far end, allowing rotation. In contrast, retractile tails decelerate based on the helices stretching and becoming a straight filament with mobility loss.^[25]^

It is possible to overcome issues arising from non‐effective particle asymmetry through asymmetric collective behavior of particles, through a nonsymmetric actuation field or a spatially asymmetric environment, i.e., a boundary, producing surface‐assisted “walking.”^[5]^

## 3 MNM Mobility Modes

Mobility is dictated by MNM compositional functionality, which determines propulsion mechanisms and can be either fuel‐dependent or independent. Fuel‐dependent MNMs are propelled by surface tension gradients, the Marangoni effect, or fuel catalysis. With fuel independence, MNMs do not require substrate addition or decomposition to move non‐reciprocally—but are dependent on external stimuli such as acoustic, electric, light, and magnetic fields. External fields allow the precise direct manipulation of programmable, reconfigurable motor systems to achieve their spatial and temporal control by tweezer action, or indirectly by enhancing directionality.

### 3.1 Fuel‐dependent Mobility

#### 3.1.1 Marangoni Flow

Chemical or temperature gradients create high energy density regions and surface tension differences at the motor‐continuous phase interface, also known as Marangoni stresses.[^27^, ^28^] This difference in surface tensions induces a fluid and chemical mass transfer down the energy gradient along the surface of the motor, bringing the system to equilibrium. Consequently, the motor moves opposite to the fluid flow to conserve the mass of the system, thus propelling the MNM, via electric fields, chemical and temperature gradients as observed with surfactant stabilized active emulsions or in photo‐thermally phoretic particles.[^29^, ^30^, ^31^]

**Figure 3:**
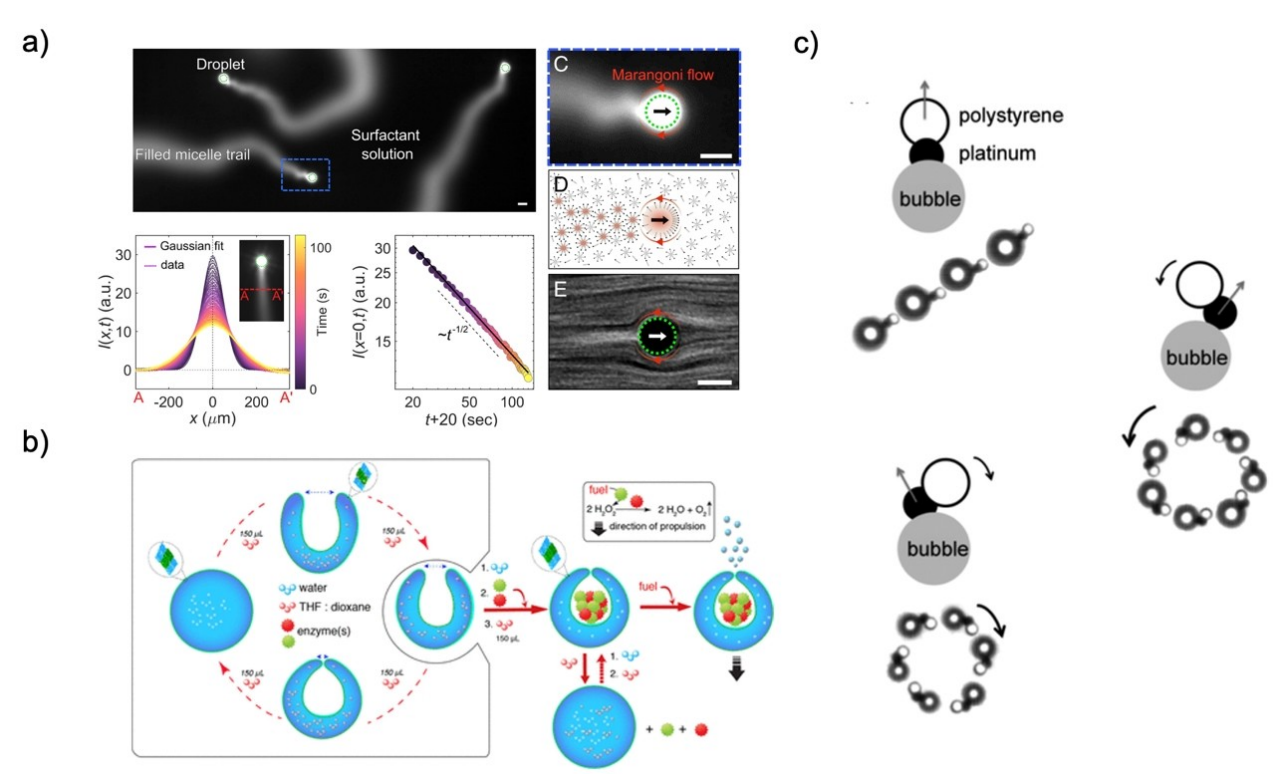
(a) Marangoni‐driven emulsion propulsion mechanism and visualization of the chemical trail. Adapted from Ref. [36]. (b) Supramolecular assembly of the enzyme‐driven stomatocyte nanomotors and propulsion mechanism. Adapted from Ref. [16]. (c) Motion behaviors of Pt‐PS hybrid dimers based on bubble orientation. Adapted from Ref. [35].

As an example, volume fractions of active emulsions are solubilized and removed by micelles in supramicellar systems, which result in surfactant gradient coverage at the motor interface and interfacial flows. The propulsion results in a micellar trail of fuel that can affect the motion of other traveling emulsions, as observed by Hokmabad et al. The trails are chemo‐repulsive, and collective motion results in negative chemotactic self‐caging (Figure 3a).^[32]^

#### 3.1.2 Chemical Decomposition

MNMs can swim by the chemical decomposition of substrates via metallic or enzymatic catalysis. Such chemical decomposition induces the formation of chemical gradients and Marangoni flows, as observed by Brooks et al. These flows can either push the motor towards higher fuel concentrations or away through repulsive Hofmeister‐type ionic interactions and electrolyte diffusion, e.g. self‐electrophoresis, to realize positive and negative chemotaxis, respectively (Figure 3b).^[33]^

The use of higher concentrations of fuel, such as H_2_O_2_, can also form local, highly concentrated areas of gas molecules, forming bubble‐driven propulsion. The force created by the growth, detachment, and collapse of bubbles can result in pressure differences and fluid displacement around motors. Subsequently, the bubble recoil imparts momentum to the structures and displaces surrounding motors, which move at high speeds and ballistically in random directions depending on their orientation around the bubble (Figure 3c).[^34^, ^35^]

Chemically fueled MNMs often have high towing force and speeds and are inherently able to find the route to the fuel source due to the favorable binding between substrates and catalysts, thus presenting themselves as relevant swimmers for targeting applications.[^33^, ^36^, ^37^]

However, issues are similar to those found in Marangoni‐induced flows, where fuel depletion or the uniform diffusion of chemicals limit autonomous movement at longer time scales. In addition, most motors are based on (toxic) fuels, limiting their applicability in biological media. More suitable motors with fuel‐independent propulsive mechanisms have been developed but lack the inherent targeting function of chemotactic motors.[^35^, ^39^]

### 3.2 Fuel‐independent Mobility

#### 3.2.1 Acoustic Actuation

**Figure 4:**
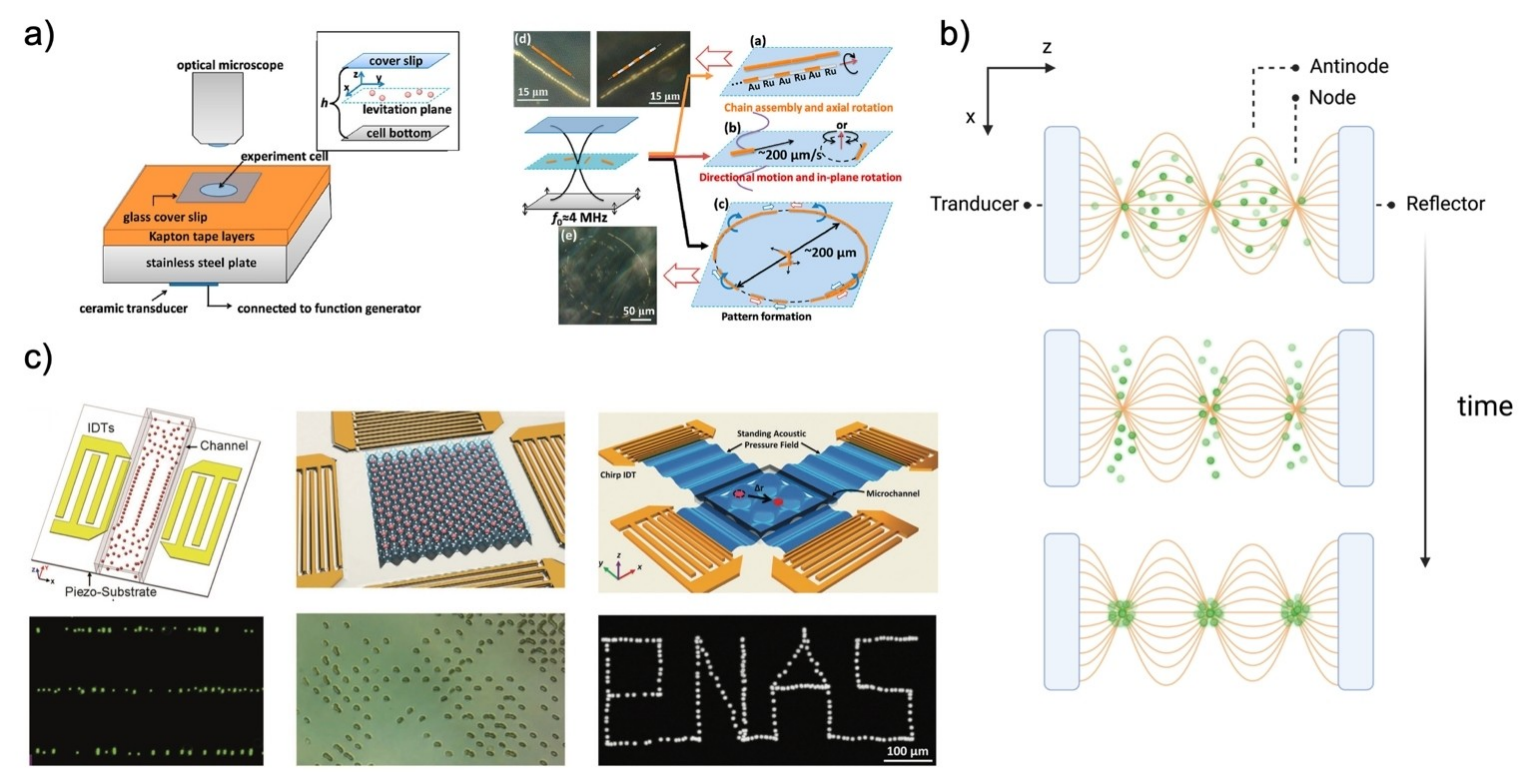
(a‐left) Microscopy chamber set‐up for acoustic actuation. (a‐right) Mobility types of bimetallic microrods. Adapted from Ref. [15]. (b) Distribution of suspended particles over time under ultrasonic standing waves. Adapted from Ref. [10]. Created with “BioRender.com”. (c) Examples of particle population manipulation by ultrasound tweezers. Reproduced from Ref. [10].

Acoustic sound waves are a non‐invasive actuation type, and movement is operated by ceramic transducers in the MHz range (Figure 4a). Through these waves, it is possible to manipulate, coordinate location, induce burst speeds in the mm s^−1^ range, and movement with no concern for ionic content, toxic fuels, and viscosity of the medium, making these motors highly interesting for biological media.[^15^, ^40^, ^41^]

The theory of ultrasonic manipulation of motors is based on the contactless absorption, scatter, and reflection of acoustic radiation forces by particles. The following equation gives the radiation on a particle at location *r*:

(1) $$F=-\left\langle V\left(t\right)\nabla p\left(r,t\right)\right\rangle =-\left(\frac{\pi p_{0}^{2}V\beta_{w}}{2\lambda }\right)\Phi \left(\beta ,\rho \right)sin\left(2kd\right)$$

Where *k* is the wavenumber, *l* the wavelength, *V* is the volume of the particle, *d* the distance between the particle and the node/antinode, *p*_0_ the pressure amplitude, and $\Phi \left(\beta ,\rho \right)$ represents the density and compressibility relationship between the particle and medium. The last‐mentioned term influences and determines the direction of the radiation force.

The colloids oscillate, rotate, and scatter radiated waves, traveling to the nearest pressure nodes and antinodes depending on the $\Phi \left(\beta ,\rho \right)$ term (Figure 4a–b).^[15]^ Here, high‐density particles, $\Phi \left(\beta ,\rho \right)$ >0, move to the nodes of a wave, whereas more flexible structures, $\Phi \left(\beta ,\rho \right)$ <0, such as liposomes, will flow to the antinodes. The scatter of the waves is directed to the neighboring particles, affecting the population‘s behavior into forming a pattern, tailorable by manipulating the relative amplitude or frequency of the pair of waves.

Surface standing acoustic waves, SSAW, are generated using interdigital transducers (IDTs), which when coupled to a piezoelectric substrate, set particles spatially, thus achieving quorum control and earning this type of control the name of acoustic tweezers (Figure 4c). Nonetheless, the scatter of the waves affects the population‘s overarching behavior, pushing research for more control in larger scale populations or for sub‐micron scaled particles. The equipment set‐up still requires ulterior quality and fabrication optimization of the IDTs and scaling to accommodate precise, three‐dimensional manipulation and reduce structural cell flaws that hamper precise wave generation and control.[^9^, ^42^, ^43^, ^44^, ^45^]

Nadal and Lauga provided a theoretical framework for the physical propulsion of microswimmers based on asymmetric fluid streaming, where the rigid bodies absorb and oscillate from radiation forces. The oscillations form stresses re‐radiated as local pressure streaming, resulting in motion perpendicular to the oscillation direction.^[18]^ However, high‐speed cameras and particle‐tracking analyses indicate additional mechanisms of motion at play for axial spinning and in‐plane rotation, potentially caused by density inhomogeneity.^[46]^

#### 3.2.2 Electrically Powered Motion

**Figure 5:**
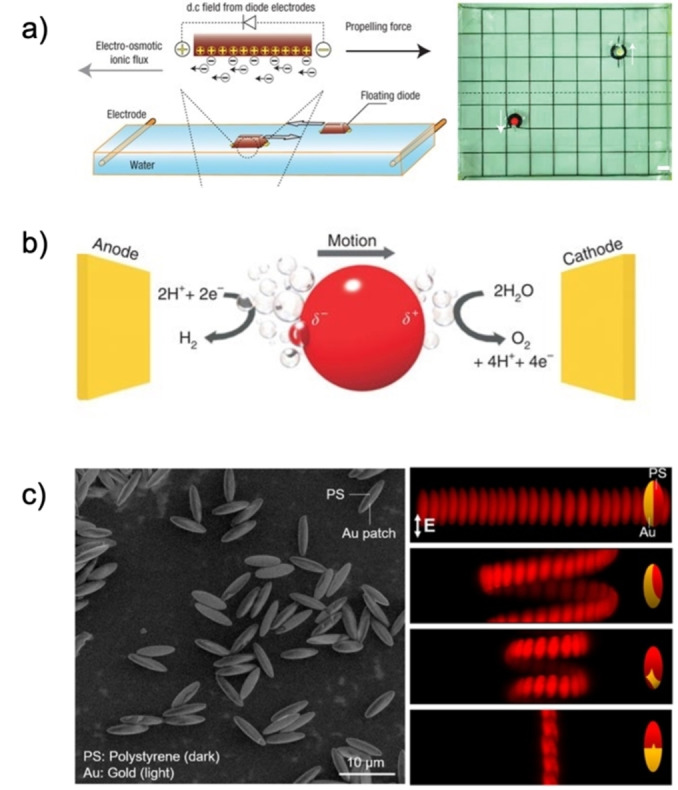
(a‐left) Electro‐osmotic flow and diode propulsion via DC fields; (a‐right) Electro‐osmotic actuation and motion of LED diodes. Adapted from Ref. [48]. (b) Mobility by bipolar electrochemical water splitting. Adapted from Ref. [50]. (c) Scanning electron micrograph of polystyrene (PS)‐Au ellipsoids and propulsion trajectories by E fields based on Au surface coverage. Reproduced from Ref. [24] under the terms of the CC BY 4.0 license.

Other examples of fuel‐free stimuli are E fields, where cells with patterned microelectrodes can induce alternating (AC) or direct current (DC) fields to manipulate E field‐responsive motors. Motion is triggered by low‐frequency fields, which apply a potential and cause an electro‐osmotic flow in the chamber. The resulting flow propels the motors into the direction of the electrodes, a process known as induced‐charge electrophoresis (ICEP) (Figure 5a and 5c).^[47]^ This MNM type can also be triggered by acting as fixed or polarized diodes creating local electro‐osmotic flows, enabling their propulsion in controlled directions depending on the orientation of their nodes, their surface charge, and internal logic (Figure 5a).[^48^, ^49^]

Finer control in motor manipulation has been found using uniform AC and DC, or electric tweezers. Applying a DC field generates electrophoretic forces, and an AC field creates a dielectrophoretic torque in the colloids, which move and reorient themselves in the longitudinal direction of the field to minimize the potential energy.[^51^, ^52^, ^53^] The intensity of these fields can also induce a maximum polarization voltage and reactions at opposite ends of catalytic metallic particles, as observed in the following equation:

(2) ΔV=E×L

Where *L* is the particle diameter, *E* the electric field, and Δ*V* the maximum polarization voltage. The last term causes reduction and oxidation reactions of water at the opposite particle poles that are oriented towards the electrodes, leading to asymmetric bubble formation from the reactions and linear or rotational propulsion depending on the motor shape.^[50]^ However, it is essential to note that a significant change in polarization voltage comes with larger motor diameters, and limitations when excessively high‐intensity fields are required to move smaller motors (Figure 5b). Moreover, electrolytes surrounding the motors may hinder the actuation of the propulsion and cause issues when motors are subjected to an *E* field. The large electric current set‐up limits the applicability in biological samples, and combining all factors indicates the need for additional studies in this area.

#### 3.2.3 Light Induced Motion

**Figure 6:**
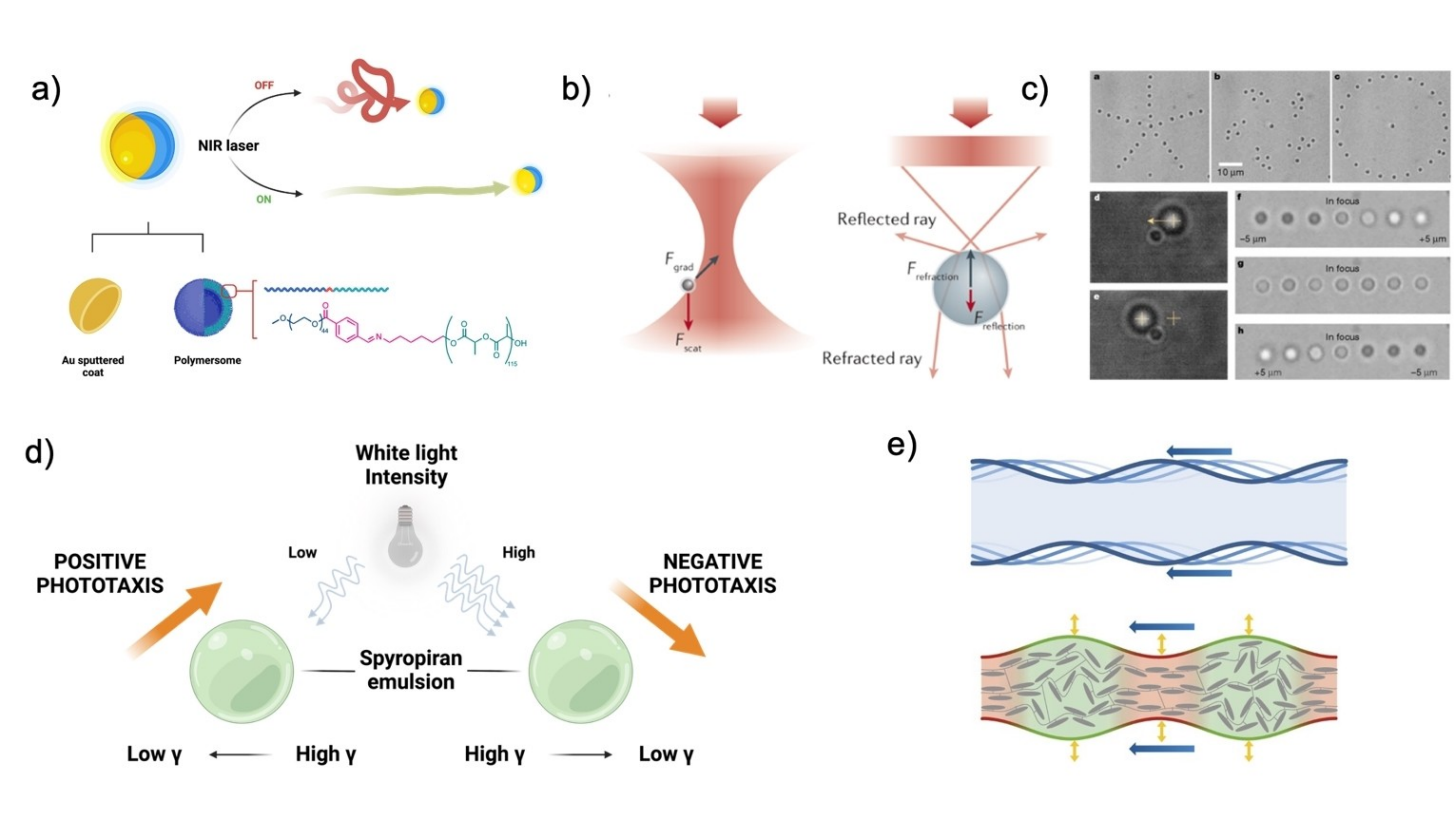
(a) Near Infrared light‐induced thermophoresis of Janus Au‐coated polymersomes. Adapted from Ref. [55] under the terms of the CC BY 4.0 license. (b) Force balances in optical trapping based on colloidal particle size. Adapted from Ref. [56]. (c) Colloid population manipulation by holographic optical tweezers. Reproduced from Ref. [57]. (d) Mechanisms for phototactic motion of photo‐responsive emulsions. Adapted from Ref. [58]. (e) Traveling wave‐like locomotion schematic of living organisms and soft microbots. Adapted from Ref. [59]. (a, d) Were created with “BioRender.com”.

Most literature comprehends light‐induced catalysis and Marangoni flow (Figure 6a).[^54^, ^55^] However, light‐driven machines can also be powered by single‐beam gradient force traps, optical tweezers, or light‐enabled traveling‐wave locopodic motion.

Optical tweezers are capable of particle trapping near the focal point of a highly focused beam via a balance of forces generated by light. The theoretical principle dictates that a converging beam generates an intensity gradient to which particles smaller than its optical wavelength, Rayleigh scatterers, can react by becoming polarized, denoted by *a* (Eq. 3), or by being a refractive object if the diameter is larger than the optical wavelength. In the first case, the beam of light induces a dipole moment in the particle at a location $\vec{r}$ and time *t*, the dipolic energy which is lowest at the highest intensity of the beam. Then, in nonhomogeneous fields generated by the beam, the particles move to the region with the highest intensity gradient of the electric field near the focal plane. The phenomenon is denoted as gradient force, ${\vec{F}}_{grad}$ (Eq. 3), which is counterbalanced by the scattering force, ${\vec{F}}_{scat}$ , caused by the absorption and scattering of light by the colloids (Eq. 4:

(3) $${\vec{F}}_{grad}=-\left\langle \left(-\alpha \vec{E}\left(\vec{r},t\right)\cdot \nabla \right)\vec{E}\left(\vec{r},t\right)\right\rangle =\;\frac{\alpha }{2}\nabla \left\langle E^{2}\left(\vec{r},t\right)\right\rangle =\frac{\alpha }{4}\nabla \left|E_{0}^{2}\right|=\left(\alpha /2cn_{m}\epsilon_{0}\right)\nabla I$$

(4) $${\vec{F}}_{scat}=\frac{n_{m}\sigma_{ext}}{c}\left\langle {\vec{S}}_{i}\right\rangle =\frac{n_{m}\left(\sigma_{abs}+\sigma_{scat}\right)}{c}\left\langle {\vec{S}}_{i}\right\rangle$$

Where c is the speed of light in vacuum, *n_m_* is the particle refraction index, $\epsilon_{0}$ is the permittivity of the vacuum, and *I* the light intensity gradient. In Eq. 4, the $\sigma_{ext}$ is the extinction cross‐section of the particle, $\sigma_{abs}$ and $\sigma_{scat}$ its absorption and scattering contribution, and $\left\langle {\vec{S}}_{i}\right\rangle$ the Poynting vector of the beam, which describes the magnitude and direction of energy flow of an electromagnetic wave (Figure 6b).^[56]^

For particles larger than the optical wavelength, these become refractive objects subject to the momentum of light and Newton's second law, where force is defined as the rate of change of momentum. As the momentum of light changes by refraction, reflection, and scattering, the particle will have an equal, oppositely signed rate of change of momentum, which balances the forces acting upon it, causing stable optical trapping. In doing so, it has been possible to directly manipulate and rotate multiple particles three‐dimensionally (Figure 6c).[^57^, ^60^, ^61^, ^62^, ^63^, ^64^]

Other options include micron‐sized, photo‐responsive liquid droplets or liquid crystal elastomeric hydrogels, which contract and swell upon traveling wave irradiation of a specific wavelength. In the former case, droplets containing photochromic molecules are positively phototactic due to the photo‐switching of the solute into a state that reduces the surface tension of the droplet. This behavior is reversed at high enough light intensity, where strong Marangoni stresses are created, and negative phototaxis is observed (Figure 6d).^[58]^ In the latter case, light can switch the elastomer from the nematic state to the isotropic state, resulting in a radial expansion and axial contraction and, ultimately, movement akin to ciliates (Figure 6e).[^59^, ^65^]

Although much focus is directed on applying tweezers in biological media and neuronal studies, drawbacks are still found in their precision and versatility, considering the need for a transparent sample for a range of wavelengths. Concerning these motors, harmful UV light is a common disadvantage that limits the range of wavelengths available for use, and poor wave penetration and high absorption from biological samples are prevalent issues. Furthermore, tweezer‐specific concerns are found in the combined inherent heating of a focused beam and materials trapped in the optical beam. In response, photo‐activated Marangoni‐driven machines or combinations of optical tweezers and other stimuli have been studied.[^66^, ^67^]

#### 3.2.4 Magnetically Powered Motors

Magnetically powered motors, also called magnetic robots (MagRobots), with translatory rolling, spinning, and tumbling motions have been developed and can be controlled with magnetic/electromagnetic (Lorentz force‐driven) fields. This locomotion type has been the object of vast scrutiny for biomedical applications due to its non‐invasive remote maneuverability and the energy transduction mechanism of the stimulus, which results in no toxicity at low field magnetic field strengths.^[5]^

Typically, magnetically powered locomotion requires a magnetic field and motors often composed of magneto‐responsive materials (ferro‐ and ferrimagnetic, paramagnetic, and superparamagnetic materials, e.g., Fe, Ni, Co, and iron oxides). The first component is generally produced by electromagnet systems of coils spatially set up around the sample, which generate different types of magnetic fields (rotating, oscillating …) upon the flow of an electric current through each coil.

A MagRobot with volume ν in an external magnetic field B will exhibit a magnetization M . In a gradient field, the motor will be subject to an attractive force (Eq. 5). In both scenarios, the machine will experience torque (Eq. 6) and align its magnetization axis (dictated by shape anisotropy) parallel to the field lines to minimize its energy:

(5) F=ν(M×∇)B

(6) T=νM×B

**Figure 7:**
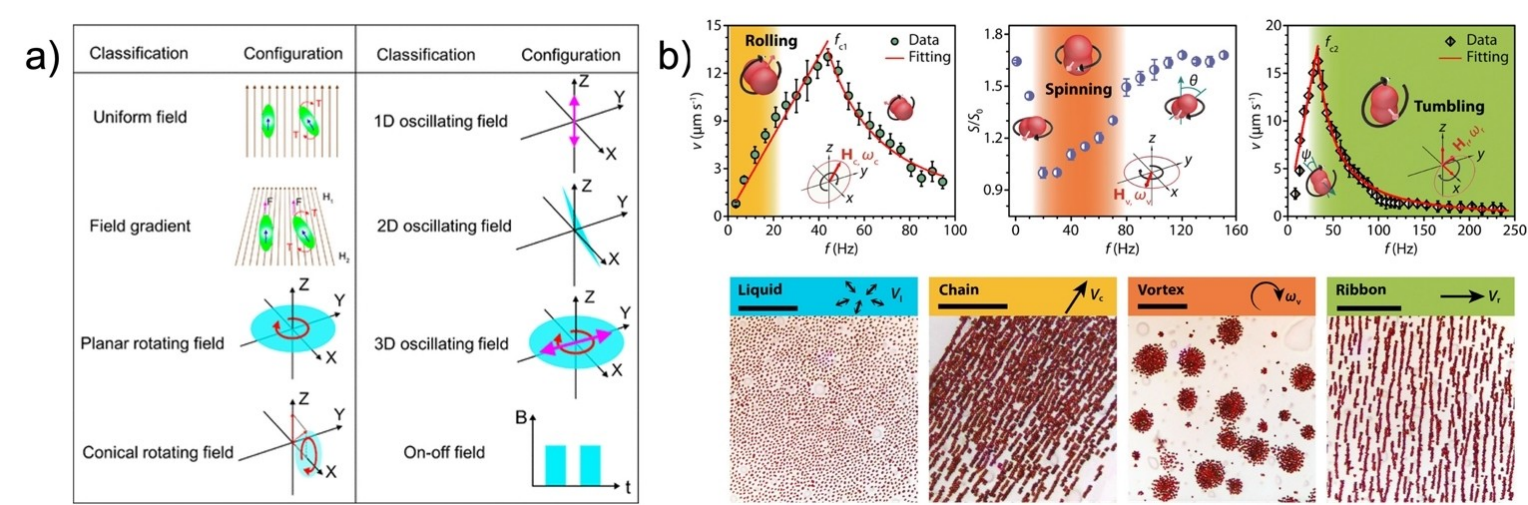
(a) Magnetic field configuration and relation to motor movement. Reproduced from Ref. [5] under the terms of the CC BY 4.0 license. (b) Propulsion mechanisms and collective behaviors of dimers in magnetic fields. Adapted from Ref. [68].

Depending on the qualities of the motor and magnetic fields, translatory and rotational movements occur with velocities proportional to the intensity of the applied field frequency (Figure 7a–b). As described in the work of Wang et al., motors tend to “wobble” at low frequencies due to the magnetization axis not aligning with the direction of the surrounding field.^[24]^ At increasing frequencies, the wobbling angle decreases from 90° to 0°, resulting in the complete alignment of the axis to the field and, ultimately, translational movement. This trend is also associated with increasing propulsion and is observed until the “step‐out frequency,” where the magnetic torque in the motor does not match the applied field and cannot maintain a synchronous relationship that leads to movement (Figure 7b).^[24]^ This limit can be regulated with surface chemistry by tuning the degree of wettability and interfacial slippage between the medium and the motor.^[69]^ Disadvantages in magnetically powered or driven machines are primarily found in the complexity of setting up the field, having appropriate motor morphology that responds to the said field, and the cost of equipment to induce the intended effects and manipulation worthy of precision medicine.

## 4 Mobility Control

Mobility control is generally hindered by thermal fluctuations, i.e., Brownian motion and rotational diffusion. Thermal molecular motions in a continuous phase randomly displace and rotate particles, thus changing their orientation over time. Therefore, asymmetric energy in‐flow, stimuli or fuel presence, and directed outflow are necessary to induce a force imbalance and a non‐equilibrium environment that allow motors to overcome these physical limitations. The energy flow causes directionality and collective dynamic mobility. This outcome is achievable through appropriate propulsion mechanisms and the inclusion of multiple “engines” within one system to produce complex behaviors.

### 4.1 Directionality Control

Typically, an active colloid within a system with uniform fuel distributions will convert the fuel, generate its chemical gradient and move directionally dictated by its surface polarity.^[70]^

**Figure 8:**
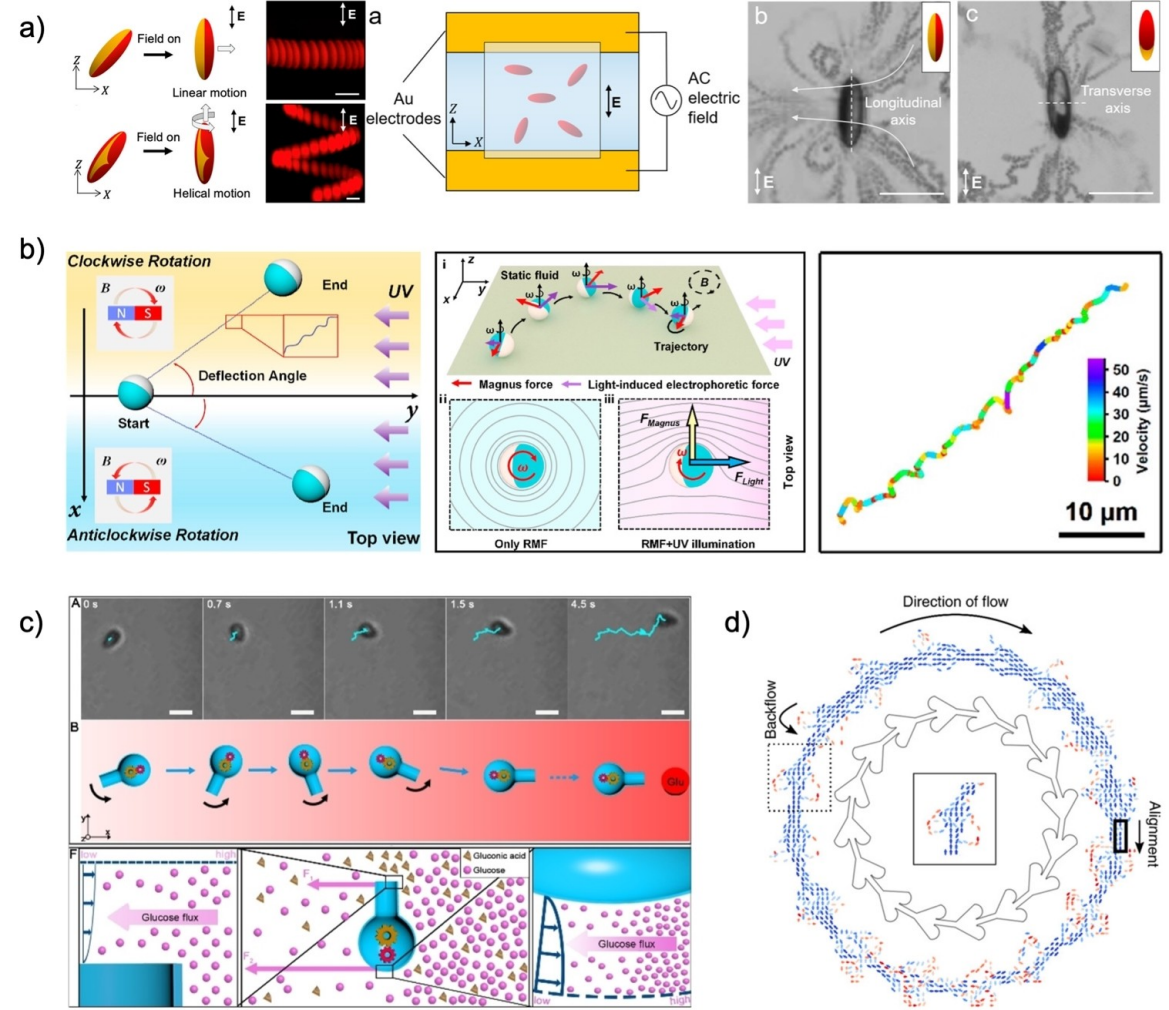
(a) PS−Au ellipsoids reorientation and propulsion trajectories in an electric field based on Au surface coverage. Adapted from Ref. [24] under the terms of the CC BY 4.0 license. (b) Schematic of the hybrid actuation system and mobility behavior of Janus micromotors under magnetic fields and directional light. Adapted from Ref. [74]. (c) Trajectory and torque‐driven reorientation mechanism of flask‐shaped motors in a chemical gradient. Adapted from Ref. [72]. (d) Janus micromotors directed flow through spatial asymmetry. Reproduced from Ref. [77].

Motion can be directed through the coordinate insertion of an equilibrium‐destabilizing element, a fuel source or external stimulus within a section of the system. In response, motors will move towards or away from the directing element, according to its active site morphology, propulsion mechanism, and thermodynamic laws, which are enthalpy or entropic diffusion‐driven.^[71]^ Concrete examples of directionality are observed with magnetic driving, where vector fields formed upon a stimulus are sensed by swimmers, and the latter respond by reorienting themselves and moving positively or negatively along the field lines (Figure 5b–8a).^[20]^

Acousto‐phoretic particles move in the directions of the wave nodes within an acoustic chamber where the potential energy is zero, chemotactic particles move along chemical gradients, E fields draw colloids in the direction of the electrodes, magnetic colloids align their magnetization axis along with the field lines, and particles will undergo forces and be trapped in optical beams. In all cases, a torque induces particle reorientation towards or away from the externally imposed input, minimizing the potential energy thusly. Such behavior becomes even more pronounced in chemotaxis with more significant motor anisotropy, where shapes are easily subject to fluid‐induced torsional forces.^[72]^ Here, flask‐shaped motors constantly reorient themselves depending on fuel concentration gradients and move in the direction of the fuel sources facilitated by their shape, which enhances chemotactic scavenging behaviors (Figure 8b). Other types of directed motion are possible via hybrid, dual‐triggered systems, where catalysis enables propulsion and physical stimuli lead the orientation of the motors (Figure 8c).^[73]^

Limitations occur predominantly in the required inputs for the actuation and propulsion mechanism. Chemotactic scavenging has been studied mostly with toxic fuels and is hindered by ionic concentrations; electric‐powered motion requires large diameter particles for electrocatalytic motion; high‐intensity light may damage samples. Therefore, combining multiple engines within a system is beneficial to induce persisting, non‐toxic, directional control, which may even allow motion direction reversal. The latter can be observed in the work of Li et al. or Shao et al. Li and co‐workers employed magnetic fields to reduce the rotational diffusion of magnetic, photo‐catalytically driven particles and increase the directionality of movement (Figure 8c).^[74]^ In Shao et al., a photo‐responsive gold coating was placed on the surface of catalase‐powered stomatocytes to enable propulsion at low H_2_O_2_ concentrations. Fuel decomposition drove stomatocytes chemotactically in one direction. Irradiation with NIR light induced thermophoretic motions that reduced stomatocyte movement and even reversed it upon switching actuation modes (Figure 6a).^[76]^ A similar concept was also reported by Liu et al.^[77]^

Directed motion can also occur by the interaction of motors with the flow fields near boundaries, otherwise known as thigmotaxis. This directionality has been shown in the catalytic colloids of Katuri et al. and is widely observed in the magnetically‐driven motor literature, such as in the work of Wang et al.[^75^, ^78^]

In the first case, the asymmetric, ratchet shape of the chamber can rectify and direct the flow of catalytic, hemispherically coated Pt Janus colloids in a specific direction (Figure 8d).^[75]^ Secondly, high friction, apparent viscosity, and contact with physical boundaries allow for net displacements of magnetic “walkers.”^[5]^ For example, magnetic microdimers tumble on the surface of walls, resulting in net displacement by traction forces (Figure 7b).[^75^, ^78^] In both cases, topographical features change the hydrodynamic stress, inducing an orientational alignment and asymmetric interfacial flows based on chamber design (i.e., high vs. low friction, roughness).

### 4.2 Rotational Control

Rotational MNM movement strongly relies on motor design coupled with actuation mode. So far, control with this type of movement has only been achieved via MNM shapes, magnetically anisotropic structures, and rotating magnetic fields.

As described in Section 2, the motor design of pinwheel shapes can direct compensating fluid flows to rotate the structures about their axis; helical structures in rotating fields display translatory motion enabled by rotation about their long axis (Figure 2d).[^14^, ^24^]

**Figure 9:**
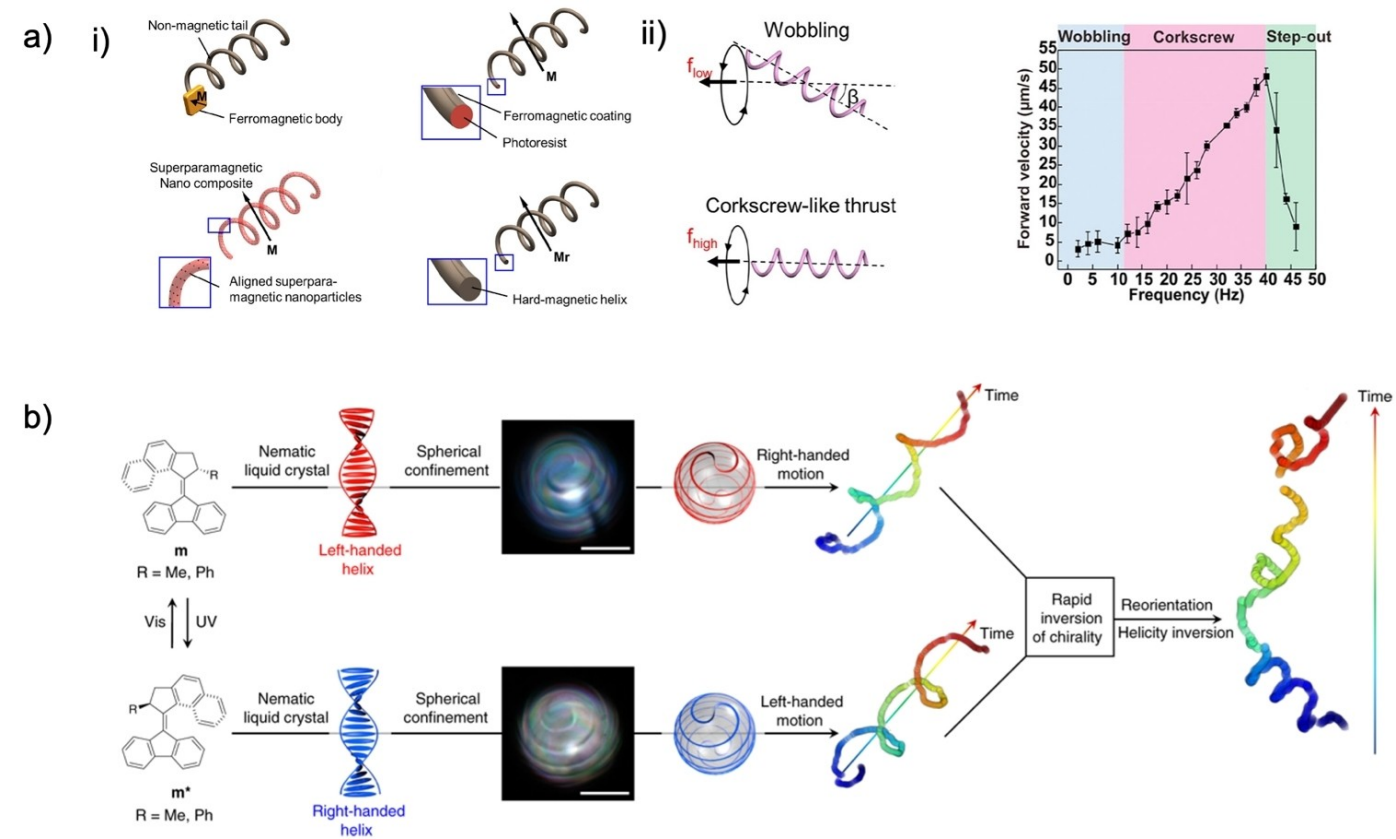
(a) Artificial helical motor structures and motion dependency. i) Common artificial rotating motor strategies. Adapted from Ref. [80]. ii) Motor velocity dependency on the magnetic field frequency. Adapted from Ref. [24]. (b) Mechanism and trajectory for helix inversion and chirality transfer from molecular machine to Marangoni‐driven emulsions. Reproduced from Ref. [31] under the terms of the CC BY 4.0 license.

Alternate strategies fall within the biomimicry of flagella or hybrid actuation forces in multi‐layered Janus spheres. In the first strategy, a flagella‐simile with a soft polymer segment and a magnetic head or tail segment rotates synchronously with the magnetic field and moves translationally like bacteria (Figure 9a).[^24^, ^79^, ^80^] In the second case, a hybrid actuation system can enable propulsion and rotation control, affecting overall mobility. Janus spheres with a magnetic core, a TiO_2_ shell, and a Pt half‐coat move with unpredictable directionality fueled by the photocatalysis of water into H_2_O_2_ and then into molecular oxygen. With low‐frequency fields, the Janus spheres spin and counteract Brownian rotational diffusion, synergistically enhancing the directionality of MNM rotation (Figure 8b).^[74]^ Alternatives in rotational movement are possible by employing spiral surface conformations that direct flows helically, as observed in Marangoni‐powered, photo‐switchable, chiral liquid crystal droplets of Lancia et al. (Figure 9b).^[31]^

### 4.3 Quorum Control

**Figure 10:**
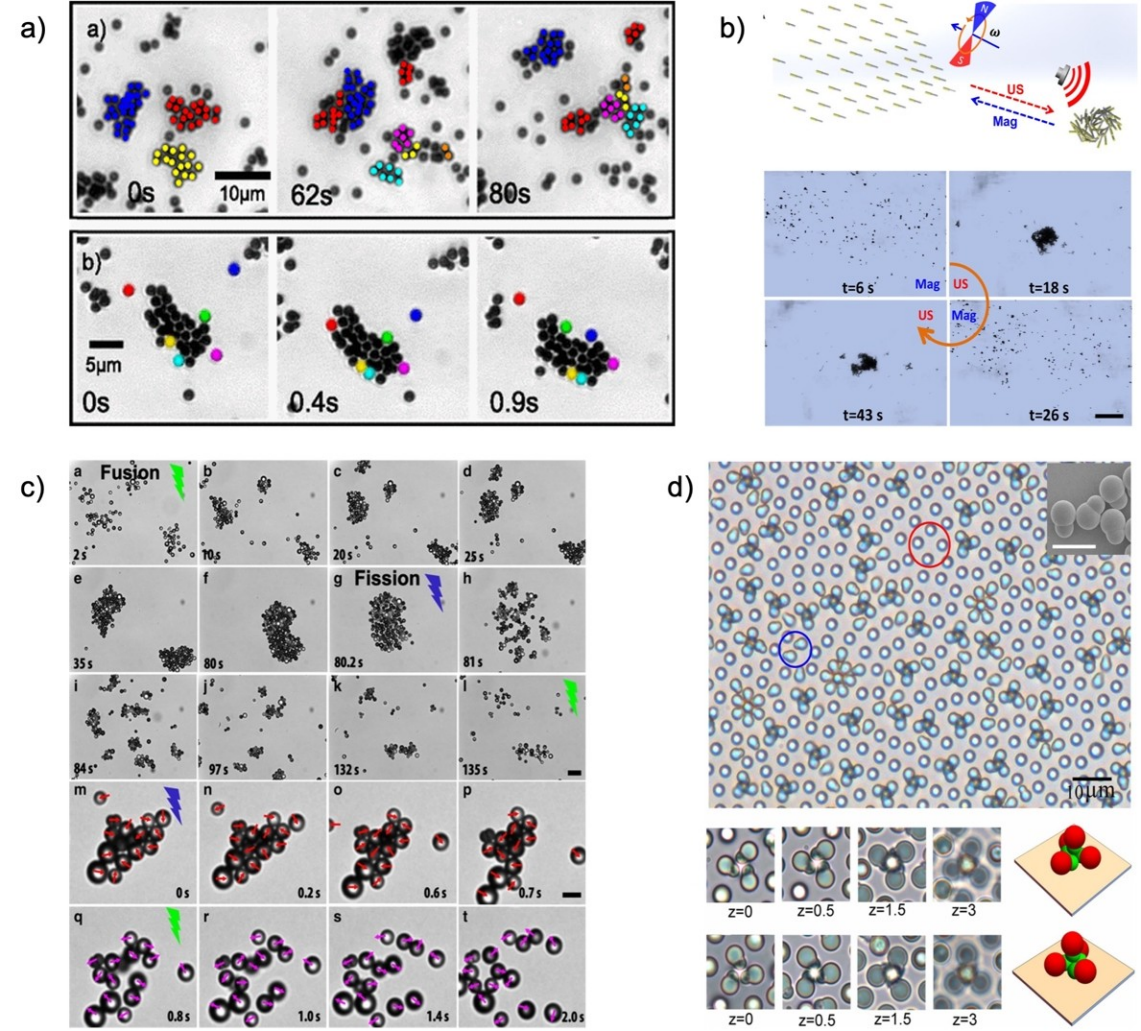
(a) Quorum dynamic motion of chemotactic spherical colloids. Reproduced from Ref. [82]. (b) Reversible behavior of magneto‐acoustic micromotors. Adapted from Ref. [84]. (c) Photocatalytic motion and behavior of Janus particles. Reproduced from Ref. [85] under the terms of the CC BY 4.0 license. (d) A‐/chiral colloidal assembly of asymmetric motors under an electric field. Adapted from Ref. [86].

Control in collective MNMs motion, or quorum behavior, can allow the remote navigation and cooperative execution of tasks impossible for individual swimmers.^[81]^ Collective movement is a phenomenon where several individuals respond to an event through coordinated motion towards or away from the event location. This function results in either tactic clustering, disassembly, or general collective migrations, and can be classified as clustered or patterned swarming. Population control is a topic with various proposed mechanisms, and dedicated reviews are available here.[^3^, ^73^] The physical principle behind this behavior falls upon the propulsion mode, where localized chemical gradients or external stimuli can induce the formation of clusters or coordinated patterns. As previously described, MNMs orient themselves and move along the vector field generated upon an “event.” Depending on the location of stimuli, colloids surrounding the source will experience the same event andminimize the energy of the system by moving toward or away from the trigger, creating motor‐dense or thin areas and emerging phenomena (Figure 10a).^[82]^

Chemically driven motors are the primary type that display clustered swarming, able to show static/dynamic clustering over time because of population density, reaction speeds, and local concentration gradients. Although the physical mechanism is not fully understood and is case‐dependent, literature proposes that self‐diffusiophoretic systems form ever‐growing static clusters over time due to the entropically‐driven osmosis and concentration gradients.^[83]^ Instead, self‐electrophoretic motors create an imbalance in ionic distribution within the electrical double layer, creating an electric field that propels them into steady‐sized dynamic groups. The assembly is dictated by the shape anisotropy and induced dipole character of the motors, whose electrostatic imbalances generate torque and dynamic alignment of colloids or electrical/osmotic repulsion. Ultimately, the combination of chemotaxis with phoresis gives rise to electrostatic and gravitational behaviors, resulting from Debye screening, microphase separation, and gravitational collapse.[^70^, ^83^]

Reversible particle assembly and disassembly is possible when multiple engines are included in the motor, where one actuation mode promotes clustering and the other disassociation (Figure 10b).^[84]^ The switching between propulsion modes changes the hydrodynamic interaction of the active particles from attractive to repulsive, or vice versa, and has been shown for photocatalytic motors. For example, photocatalytic Janus colloids presenting a titania hemisphere, responsive to blue light, and a gold one, responsive to green light, have been shown.^[85]^ These spheres cluster due to the photocatalytic activity of the gold coat, whose products induce solute‐mediated diffusiophoresis and particle clustering via long‐range attractions. The catalytic activity of the TiO_2_ area is higher than the one of the gold, and repulsive osmotic imbalances are formed in clusters that disassociate the groups by self‐diffusion electrophoresis (Figure 10c).[^83^, ^85^] These collective oscillations have been widely discussed by Golestanian and co‐workers.^[70]^

Aside from the manipulation tactics given by optical and magnetic trapping, gradient physical stimuli create vector fields to which identical particles respond similarly, forming synchronous collective displays programmed by the manipulation of the field lines. MNM‐dense areas are driven by vector field actuation, leading to quorum dynamic or static phenomena. Dipole‐induced motors, electric or magnetic, can align themselves along field lines, and their poles can interact with the opposite ends of other MNMs, forming static and dynamic supracolloidal assemblies, as observed in magnetic dimers and rotating fields or asymmetric polymeric dimers in electric fields (Figure 10d).[^5^, ^86^, ^87^]

### 4.4 Speed Regulation

**Figure 11:**
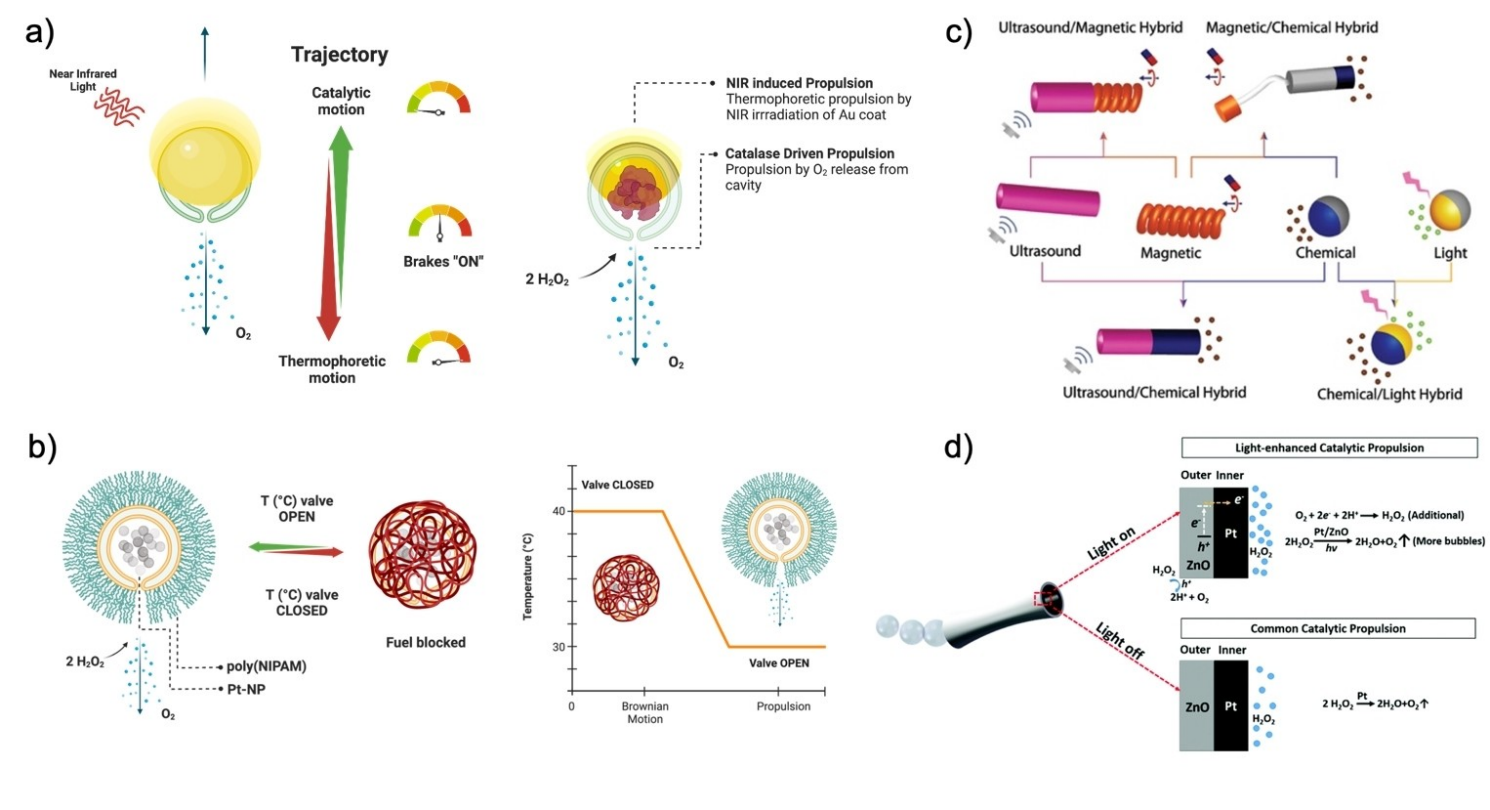
(a) Twin engine motion of Au‐coated stomatocytes filled with catalase. Adapted from Ref. [76] under the terms of the CC BY 4.0 license. (b) Stomatocytes coated with temperature responsive polymers acting as a fuel blockage plug. Adapted from Ref. [88]. (c) Examples of hybrid MNMs. Reproduced from Ref. [73]. (d) Mechanism of microrocket propulsion with and without UV irradiation. Reproduced from Ref. [89]. (a, b) Were created with “BioRender.com”.

ON/OFF speed control is achieved with the halt of external field stimuli or with a delicate balance of forces; available strategies can be subdivided into two main categories: speed decrease or increase. Speed decrease is observed in magnetic, light, and acoustic actuation or when polymers block the fuel path to the catalyst site.[^88^, ^90^] Halting behaviors have been vastly shown with removing external acoustic, light, or magnetic stimuli. In contrast, catalyst‐powered speed decrease is possible with force balancing between motor engines, as observed in Shao's multi‐engine stomatocytes (Figure 11a), or with braking systems based on blockage of the pathway to the catalyst (Figure 11b).^[88]^

Instead, speed increase requires the synergistic action of multiple engines within a motor to reduce rotational diffusion or direct forces in the same direction, resulting in a net vector larger than the propulsion vector of the MNM. The net force supersedes Brownian motion and random walks at longer time scales. Several non‐chemical fuel‐related engine combinations use motor design to achieve the direction of forces, ranging from acoustophoretic or light‐responsive components, magnetic materials with photocatalytic propulsion, and ultrasound/magnetic hybrids (Figure 11c).[^74^, ^84^, ^91^]

Chemical fuel‐related cases employ the combination of a catalyst and an external stimulus‐sensitive component that can form fuel precursors or enhance catalytic rates, such as temperature. The most optimal and quickly actuating strategies include light‐sensitive features that increase local concentrations of fuel in the motor. For example, light induces the formation of fuel precursors via zinc oxide in ZnO/Pt microrockets. The activity of ZnO outer layer catalysis provides in situ electrons and molecular oxygen to the inner Pt layer of the rocket, which converts the precursors to H_2_O_2_ and consumes the peroxide enabling enhanced catalytic, bubble‐driven propulsion compared to the action of Pt only (Figure 11d).^[89]^

## 5 Summary and Outlook

The state‐of‐the‐art in MNM systems offers many options for motion control. Based on mechanisms of motion, directionality control is best achieved by physical field tweezers, optical, magnetic, or acoustic, or by photo‐thermally phoretic motors, which display high maneuverability, speed control, and the ability to swim in viscous media with different field strength inputs.^[92]^ High population manipulation is also achieved by the same motors, given the quorum response of motors to physical fields. A special mention is given to efficient chemotactic scavengers, which serve as a targeting function highly sought after for precision medicine. However, these MNMs require additional fuels and have low speeds compared to fuel independent options, thus limiting their applicability in concentrated environments.^[92]^ Effective rotational control has been shown using rotating magnetic fields and motor structures that inherently move by rotations, and by clever compositional design.

Significant issues must be addressed to realize the application potential of MNM systems. Clear goals and suitable modes of actuation are necessary to ensure motor mobility, cell penetration, and delivery of cargos; an appropriate motor design ensures valid applicability, and three critical aspects need to be considered and are part of the current focus of MNMs for biomedical applications, namely biocompatibility and safety of motors and substrates, mobility in physiological conditions, and cell uptake or penetration ability. Surfactant and chemically‐powered colloids often cannot be used due to the need for toxic, high concentration solutions to move; moreover, surfactant‐induced Marangoni flows are not biomedically applicable due to biocompatibility, and most basic chemical decomposition mobility systems rely on reactions with unreasonable substrate concentrations or the use on agents to reduce metal active site passivation. However, chemically decomposing engines can be beneficial for applications where the synergistic, che motactic activity of multiple catalysts, surface functionality, and byproduct formation can enhance cell uptake or treat diseases, e.g., glucose oxidase‐loaded nanomotors for crossing the blood‐brain barrier moving towards high glucose concentrations or urease functionalized motors for treating bladder‐related illnesses.[^93^, ^94^] Instead, physically‐powered MNMs made of biocompatible materials represent a valuable option for effective swimming in complex media with low toxicity inputs, and the current focus should be placed on cargo loading and delivery. On this front, fuel independent and dependent combinations and biohybrid systems based on motile living cells and physical field guiding are the focus of the MNM world.

Challenges are found in large scale MNM production, understanding the mechanism of motions with the different actuation modes, accurate and precise population control, and in finding effective combinations of multiple engines and stimuli‐responsive materials. The success of the latter plus the optimization of operating equipment will enable large‐scale motor engineering with full motion control suitable for biomedical or environmental applications.

## Conflict of interest

The authors declare no conflict of interest.

## Biographical Information

*Alexander Deen Fusi is a Ph.D. candidate working in the Bio‐Organic Chemistry group of Prof. Jan van Hest. He received his B.Sc. degree in Biotechnology from the State University of New York, College of Environmental Science and Forestry (USA). After that, he has travelled to Sweden and Finland to obtain his MSc in the N5T (Nordic 5 Technology) “Polymer Technology, Biomaterials mobility track” joint MSc program at KTH, Royal Institute of Technology, and Aalto University. Current research interests are focused in exploring the world of nanomotors and artificial organelles*.

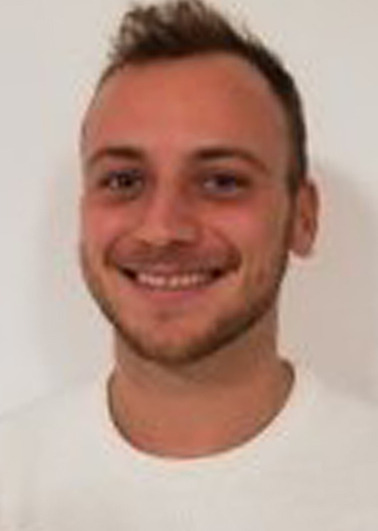

## Biographical Information

*Yudong Li received his B.Eng. degree in biomedical engineering from Zhejiang University (China), where he focused on the development of polymeric materials for wound healing, under the supervision of Prof. Ming‐Wei Chang. Afterwards, he worked on point‐of‐care testing for infectious disease detection at Brigham and Women's Hospital, Harvard Medical School (USA). Then, he started his MRes project at Imperial College London (UK) and worked on the preparation of photoresponsive polymersomes via polymerization‐induced self‐assembly supervised by Prof. Molly M. Stevens. Currently, he is a Ph.D. candidate at Eindhoven University of Technology, engineering functional polymeric assemblies for nanomedicine under the supervision of Prof. Jan C. M. van Hest & Dr. Loai K. E. A. Abdelmohsen*.

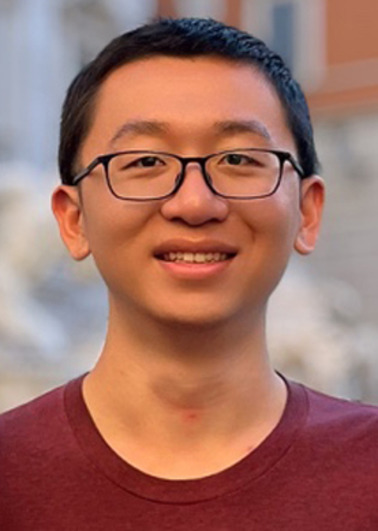

## Biographical Information

*Antoni Llopis‐Lorente graduated in Chemistry (2013) from University of Valencia, including a research internship at Imperial College London. He obtained his Ph.D. in nanotechnology at the Polytechnic University of Valencia (UPV) in 2019. He has been a visiting researcher at Complutense University of Madrid (2015), Radboud University (2017), and Institute of Bioengineering of Catalonia (2017). In 2019, he joined the Bio‐Organic Chemistry group at Eindhoven University of Technology under the research line of Prof. Abdelmohsen and Prof. van Hest. Since 2022, he is a doctor research associate at UPV. His research interests include the development of smart nanodevices and artificial cells*.

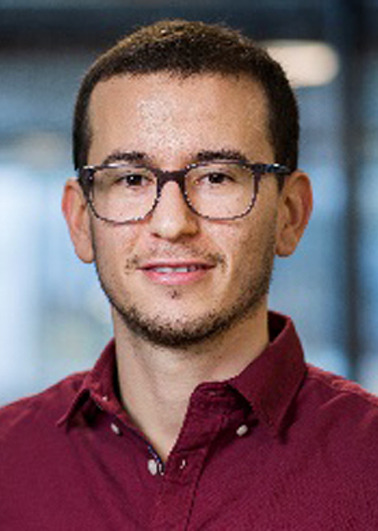

## Biographical Information

*Tania Patino obtained her Ph.D. in cell biology from the Autonomous University of Barcelona (2015). In 2016 she obtained a Juan de la Cierva postdoctoral fellowship and joined Prof. Samuel Sánchez's group at the Institute for Bioengineering of Catalonia, where she worked on the development of enzyme‐powered biomedical micro‐ and nanorobotics. In 2019, she obtained a Marie Sklodowska Curie fellowship and joined Prof. Francesco Ricci's lab at the University of Tor Vergata, Rome, where she worked on DNA nanotechnology to provide new functionalities to enzyme‐powered nanoswimmers. Since January 2022, she is a Tenure‐Track Assistant Professor at the Biomedical Engineering Department of the Eindhoven University of Technology and the Institute for Complex Molecular Systems (ICMS). Her research focuses on understanding the interactions between artificial active matter and living cells ant their potential application in cancer theranostics and immunotherapy*.

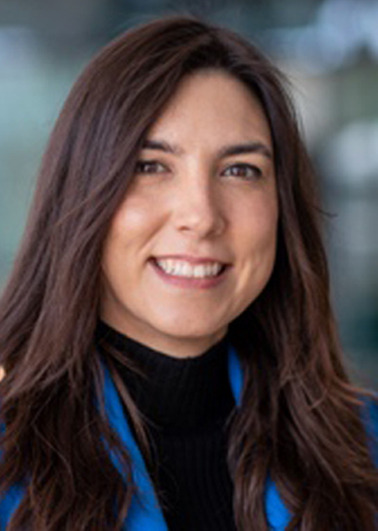

## Biographical Information

*Jan van Hest obtained his Ph.D. from Eindhoven University of Technology (1996) in macro‐organic chemistry with prof E. W. Meijer. He worked as a postdoc with prof D. A. Tirrell on protein engineering. In 1997, he joined the chemical company DSM in the Netherlands. In 2000, he was appointed full professor in Bio‐organic chemistry at Radboud University Nijmegen. As of September 2016, he holds the chair of Bio‐organic Chemistry at Eindhoven University of Technology. Since May 2017, he is the scientific director of the ICMS. The group's focus is to develop well‐defined compartments for nanomedicine and artificial cell research. Using a combination of techniques from polymer science to protein engineering, well‐defined carriers and scaffolds are developed for application in e.g. cancer treatment, immunology and ophthalmology*.

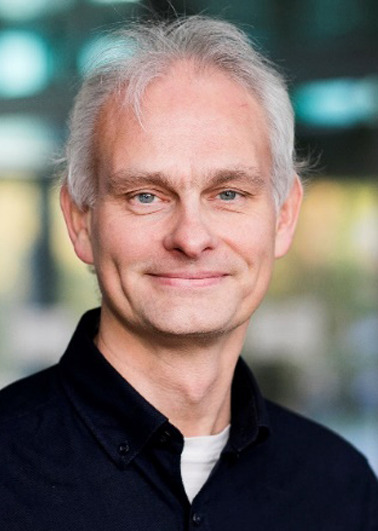

## Biographical Information

*Loai Abdelmohsen is assistant professor in the Department of Chemical Engineering and Chemistry at Eindhoven University of Technology in the Netherlands. He conducted his Ph.D. in the Bio‐Organic Chemistry group, Radboud University Nijmegen. During his Ph.D. he gained an active interest in the utilization of copolymers for supramolecular assembly and the subsequent integration of functional properties, such as motility. In 2017, after a short postdoc at Eindhoven University, he was promoted to assistant professor, leading a team of Ph.D. students and Postdocs. So far, he supervised 7 Ph.D. students, to whom he was a coporomotor. His research is focused on translating life‐like behaviors, such as motility, toward synthetic functional polymeric structures*.